# Correlation between cerebral small vessel disease and postural instability/gait difficulty subtype in Parkinson’s disease patients

**DOI:** 10.3389/fnagi.2025.1686214

**Published:** 2025-12-02

**Authors:** Yingchao Ge, Wa Zhao, Xiaorong Xu, Wenchao Qiu, Peiting Liu, Minghui Zhao, Yongqing Cheng, Shouru Xue

**Affiliations:** 1 Department of Neurology, The First Affiliated Hospital of Soochow University, Suzhou, Jiangsu, China; 2 Department of Neurology, Qidong Hospital Affiliated to Nantong University, Nantong, Jiangsu, China; 3 Department of Neurology, The Second Affiliated Hospital of Xinjiang Medical University, Urumqi, Xinjiang, China; 4 Department of Neurology, The Yancheng Clinical College of Xuzhou Medical University, The First People’s Hospital of Yancheng, Yancheng, Jiangsu, China; 5 Department of Neurology, Huai'an Hospital Affiliated to Xuzhou University, Huai'an, Jiangsu, China; 6 Department of Neurology, Chongzhou People's Hospital, Chongzhou, Sichuan, China

**Keywords:** cerebral small vessel disease, postural instability/gait difficulty, Parkinson’s disease, imaging biomarker, vascular pathology

## Abstract

**Background:**

Postural instability/gait difficulty (PIGD) subtype in Parkinson’s disease (PD) portends poorer prognosis and limited treatment response. While cerebral small vessel disease (CSVD) is implicated in motor impairment, its specific association with PIGD remains underexplored. This study aims to investigate the correlation between the severity of CSVD and PIGD subtype in PD patients.

**Methods:**

This cross-sectional study enrolled 161 PD patients (mean age 71.14 ± 7.04 years). Motor subtyping [postural instability and gait difficulty (PIGD)/tremor-dominant (TD)/intermediate type (IT)] used MDS-UPDRS-derived ratios. CSVD burden was quantified via two validated MRI-based scores: the total CSVD burden score (range 0–4) and modified CSVD burden score (range 0–6). Multivariate logistic regression analysis, which adjusted for age, gender, disease duration, and vascular risk factors, was used to explore the correlation between CSVD burden and PIGD.

**Results:**

The prevalence of PIGD was 49.07% (*n* = 79). PIGD patients exhibited a significantly higher CSVD burden than the TD and IT groups (total score: 1.84 ± 0.72 vs. 1.4 ± 0.59 vs.1.15 ± 0.49; modified score: 2.55 ± 1.20 vs. 1.85 ± 0.7 vs. 1.75 ± 0.91, both *p* < 0.001). After full adjustment, each 1-point increase in total CSVD burden score associated with 7.16-fold higher PIGD odds (aOR = 7.16, 95%CI = 1.64–30.82, *p* = 0.009), and each 1-point increase in modified CSVD burden score associated with 6.03-fold higher PIGD odds (aOR = 6.03, 95%CI = 3.06–11.90, *p* < 0.001).

**Conclusion:**

Global CSVD burden was independently associated with the occurrence of PIGD in PD. CSVD assessment may help identify PD patients at the highest risk for axial motor disability, highlighting the convergence of vascular and neurodegenerative pathologies.

## 1 Introduction

Parkinson’s disease (PD) is a clinically heterogeneous neurodegenerative disorder characterized not only by cardinal motor features but also by diverse non-motor symptoms (Pantoni, 2010). The most widely used classification for PD motor symptoms is based on three motor phenotypes: tremor-dominant (TD), postural instability gait disorder (PIGD), and intermediate (IT) types, according to the Movement Disorder Society Unified Parkinson’s Disease Rating Scale (MDS-UPDRS) (Cannistraro et al., 2019; Khan et al., 2006). Among the motor subtypes, the PIGD subtype represents a particularly debilitating phenotype, characterized by prominent axial motor impairments including freezing of gait, postural instability, and frequent falls. This subtype is associated with accelerated disease progression, poorer response to dopaminergic therapy, increased risk of dementia, higher caregiver burden, and significantly reduced quality of life compared to the tremor-dominant subtype (Khan et al., 2006; Cannistraro et al., 2019).

Concomitantly, cerebral small vessel disease (CSVD), a common age-related vasculopathy affecting the brain’s small arteries, arterioles, capillaries, and venules, manifests as white matter hyperintensities (WMH), lacunes, cerebral microbleeds (CMBs), and perivascular spaces on neuroimaging (Brown et al., 2018). CSVD burden is a well-established contributor to vascular cognitive impairment and dementia. Critically, emerging evidence suggests a significant interplay between CSVD and PD pathology. Epidemiological studies indicate a higher prevalence of vascular risk factors and imaging markers of CSVD in PD patients compared to age-matched controls (Wan et al., 2022). Moreover, CSVD burden has been linked to worse overall motor severity, accelerated cognitive decline, and potentially a higher incidence of PD itself (Mao et al., 2022).

The relationship between CSVD and specific PD motor phenotypes, particularly the PIGD subtype, is of intense interest but remains incompletely defined. Pathophysiologically, both processes may converge to disrupt critical neural circuits: PD primarily affects the basal ganglia-thalamocortical loops, while CSVD predominantly damages subcortical white matter tracts and deep grey matter nuclei. This co-occurrence could synergistically impair complex motor functions reliant on intact fronto-striatal and cortico-ponto-cerebellar networks, which are essential for balance, gait coordination, and postural control (Hosoya et al., 2024; Wan H., et al., 2023; Wan S., et al., 2023; Duering et al., 2023; Righart et al., 2013). Preliminary clinical observations and a limited number of cross-sectional studies suggest that higher CSVD burden, particularly extensive WMH and lacunes in strategic locations, may be associated with more severe postural instability and gait disturbances in PD (Oveisgharan et al., 2021; Dadar et al., 2020a). However, these studies often focus on individual CSVD markers or lack rigorous subtyping, and crucially, the potential of CSVD burden as a predictive biomarker for the PIGD subtype has not been systematically investigated.

Therefore, we conducted this cross-sectional study to explore the potential correlation between the severity of CSVD assessed by the total CSVD burden score and the occurrence of PIGD subtypes in PD patients.

## 2 Materials and methods

This was a single-center, cross-sectional observational study conducted at the First Affiliated Hospital of Suchow University between September 2020 and December 2022. The study protocol was approved by the Institutional Review Board/Ethics Committee of the First Affiliated Hospital of Soochow University, and all participants provided written informed consent.

### Participants

The inclusion criteria included (1) diagnosis of idiopathic PD according to the International Parkinson and Movement Disorder Society (MDS) Clinical Diagnostic Criteria in 2015, (2) age ≥ 50 years, (3) ability to undergo brain magnetic resonance imaging (MRI) without contraindications, and (4) ability to provide informed consent and complete the clinical assessments. The exclusion criteria were as follows: (1) atypical parkinsonism or secondary parkinsonism (e.g., vascular parkinsonism, drug-induced), (2) major psychiatric disorders or severe cognitive impairment precluding reliable assessment, (3) other diseases with MRI abnormalities, such as cerebral ischemic stroke, cerebral hemorrhage, brain tumors, brain trauma, multiple sclerosis, central nervous system infection, hydrocephalus, or autoimmune encephalitis, (4) with other diseases that can cause motor dysfunction (e.g., cerebrovascular disease, neuromuscular junction disease, limb fracture), and (5) with incomplete clinical or imaging data.

### Baseline data collection and clinical assessment

The baseline data, including demographic data (age, gender, and education level), medical history (hypertension, diabetes mellitus, hyperlipidemia, smoking and drinking history, history of stroke/TIA), and disease duration of PD, were collected on admission using a standardized protocol.

All participants underwent a detailed clinical evaluation by a movement disorders specialist blinded to the MRI findings. The Movement Disorder Society Unified Parkinson’s Disease Rating Scale (MDS-UPDRS) was used to assess motor function in the practical “off” state. Different motor symptoms were evaluated under four indicators: rigidity (neck, bilateral upper and lower limbs; section 3.3), bradykinesia (finger-tapping test, fist-making test, alternating movements test, toe-tapping exercise, leg agility, standing balance test, and global bradykinesia; sections 3.4–3.9, 3.14), tremor (postural tremor of the upper limbs, kinetic tremor, resting tremor amplitude in limbs, lips and mandible; sections 3.15–3.18), and gait/postural instability (gait, freezing of gait, pull test; sections 3.10–3.13). Each item was scored from 0 to 4, with higher scores indicating more severe symptoms.

PD was classified into three types using the ratio method (tremor score/postural gait score). A ratio greater than 1.5 was classified as TD, less than 1.0 as PIGD, and between 1.0 and 1.5 as IT (Khan et al., 2006).

### MRI acquisition and CSVD assessment

Brain MRI was performed on a 3.0 T SignaHDX MRI scanner (produced by General Electric Company) using standardized protocols, including: T1-weighted imaging, T2-weighted imaging, fluid-attenuated inversion recovery (FLAIR), susceptibility-weighted imaging (SWI) and diffusion-weighted imaging (DWI) (Brown et al., 2018) (Supplementary Table 1). MRIs were reviewed independently by two experienced neuroradiologists blinded to the clinical data. Discrepancies were resolved by consensus. White matter hyperintensities (WMH), including the periventricular and deep WMH, were evaluated based on T2 and Flair sequences by the Fazekas rating scale (Markus and de Leeuw, 2023) (Supplementary Table 2). Cerebral microbleeds (CMBs) were defined as round or ovoid hypointense lesions measuring 2–10 mm in diameter on susceptibility-weighted imaging (SWI). Lacunae were characterized as small, fluid-filled cavities located in the cortex, displaying signal intensities similar to cerebrospinal fluid (CSF) on T1, T2, or FLAIR weighted images, with diameters ranging from 3 to 20 mm (Cannistraro et al., 2019). Enlarged perivascular spaces (EPVS) were described as linear, round, or oval structures less than 3 mm in diameter, exhibiting CSF-like signal intensity (Shi and Wardlaw, 2016). These are predominantly observed in the basal ganglia and subcortical white matter regions (Supplementary Table 3).

The severity of CSVD was assessed using two scoring systems: the total CSVD burden score proposed by Staals et al. and the modified total CSVD burden score developed by Lau et al. (2017) (Staals et al., 2014; Lau et al., 2017; Wardlaw et al., 2013). Both scoring systems incorporate the four MRI markers of CSVD mentioned above. The original total CSVD burden score ranges from 0 to 4, with one point assigned for the presence of each of the following: lacunae, CMBs (each counted separately), moderate-to-severe white matter hyperintensities (WMH), defined as periventricular WMH with a Fazekas score of 3 or deep WMH with a Fazekas score ≥2, and moderate-to-severe basal ganglia EPVS (BG-EPVS) with a count of ≥11 (Supplementary Table 4). In contrast, the modified total CSVD burden score ranges from 0 to 6. One point is assigned for the presence of lacunae, CMBs with a count of 1–4, severe BG-EPVS (>20), and moderate WMH burden (total WMH grade 3–4), whereas two points are allocated for high CMB burden (≥5) and severe WMH burden (total WMH grade 5–6) (Liu et al., 2024) (Supplementary Table 5).

### Statistics

Statistical analyses were performed using SPSS version 23.0. A two-tailed *p*-value < 0.05 was considered statistically significant. Continuous variables were presented as mean ± standard deviation (SD). Categorical variables were presented as frequencies (percentages). Differences between PIGD vs. TD vs. IT subgroups were tested using Student’s *t*-test, Kruskal–Wallis test, chi-square test, or Fisher’s exact test, as appropriate.

Univariate binary regression analysis was used to screen the risk factors of PIGD, and multivariate binary regression analysis was used to explore the independent correlation between CSVD and PIGD. To adjust for confounding factors, three models were set up for regression analysis: Model 0: no adjustment; Model 1: adjusted for gender and age; Model 2: further adjusted for variables with *p* < 0.1 in the univariate regression analysis on the basis of Model 1. The goodness-of-fit of the logistic regression models was assessed using the Hosmer-Lemeshow test, which indicated a good fit (*p* > 0.05). The discriminatory power of the models was evaluated by the area under the receiver operating characteristic (ROC) curve (AUC). An association was indicated as the odds ratio (OR) or adjusted odds ratio (aOR) with the 95% confidence interval (CI).

## 3 Results

### Comparison of characteristics between PIGD and non-PIGD subgroups

**Figure 1:**
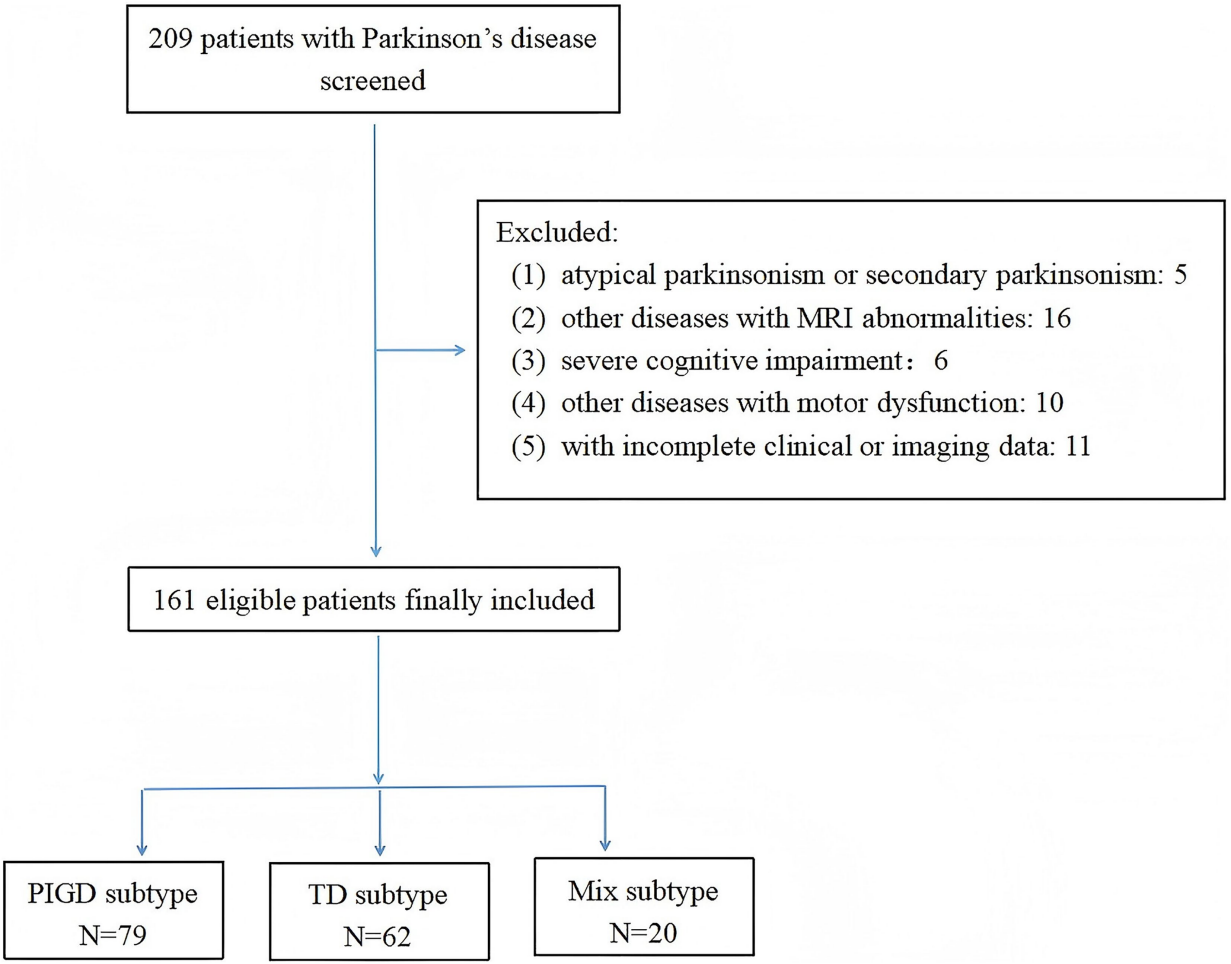
Flow chart of patient recruitment.

A total of 209 consecutive PD patients were screened. After applying exclusion criteria (*n* = 48: 5 for atypical Parkinsonism or secondary Parkinsonism, 16 for other diseases with MRI abnormalities, 6 for severe cognitive impairment, 10 for other diseases with motor dysfunction, 11 with incomplete clinical or imaging data), 161 patients were included in the final analysis (Figure 1). Participants had a mean age of 71.14 ± 7.04 years, and 41.61% were male. The mean disease duration was 4.48 ± 1.52 years. Motor severity assessed by MDS-UPDRS-III was 22.25 ± 3.65. The mean total CSVD burden score and modified CSVD burden score were 1.68 ± 0.69 and 2.55 ± 1.20, respectively. The PIGD subtype was identified in 49.07% (*n* =  79) of participants.

Significant differences were observed between PIGD (*n* =  79), TD (*n* =  62), and IT (*n* =  20) subgroups across multiple domains, including age, disease duration, UPDRS III, total CSVD burden score, modified total CSVD burden score, and proportion of DM and drinking (Table 1). Furthermore, the results indicated that compared with the PIGD subgroup, the TD subgroup has lower levels of age, PD duration, UPDRS III, total CSVD burden score, and modified CSVD burden score, a higher proportion of DM, and a lower proportion of LI. While the IT subgroup has lower levels of UPDRS III, total CSVD scores, modified total CSVD burden score, and a lower proportion of LI.

**Table 1:** Demographic and clinical characteristics according to the subtype of PD.

Characteristics	TD (*n* = 62)	Mix (*n* = 20)	PIGD (*n* = 79)	*p*
Demographics				
Male, *n* (%)	27 (63.0)	12 (67.3)	28 (66.3)	0.128
Age, mean (SD) (years)	68.49 (6)^a^	72.92 (6.28)	72.77 (7.41)	<0.001
Education, mean (SD) (years)	6.56 (2.53)	6.35 (1.98)	5.94 (2.59)	0.329
Medical history, *n* (%)				
Hypertension	16 (25.81)	6 (30)	15 (18.99)	0.461
Diabetes mellitus	13 (20.97)^a^	2 (10)	5 (6.33)	0.031
Dyslipidemia	13 (20.97)	1 (5)	11 (13.92)	0.198
Smoking	12 (19.35)	5 (25)	15 (18.99)	0.827
Drinking	14 (22.58)^a^	1 (5)	7 (8.86)	0.030
Clinical characteristics of PD				
Disease duration, mean (SD) (years)	3.82 (1.52)^a^	5.24 (1.7)	4.8 (1.27)	<0.001
UPDRS III, mean (SD)	18.61 (2.04)^a^	22.05 (2.11)^a^	25.15 (1.99)	<0.001
Imaging characteristics				
Deep-WMH Fazekas, mean (SD)	1.03 (1.09)	0.9 (0.85)	1.24 (1.08)	0.315
Presence of LI, *n* (%)	19 (30.65)^a^	4 (20)^a^	43 (54.43)	0.002
Presence CMBs, *n* (%)	23 (37.10)	5 (25)	36 (45.57)	0.211
PV-WMH Fazekas, mean (SD)	0.84 (1.03)	0.95 (1.05)	1.01 (1.02)	0.607
BG-EPVS, mean (SD)	1.89 (1.19)	1.7 (1.17)	1.85 (1.16)	0.824
Total CSVD score, mean (SD)	1.40 (0.59)^a^	1.15 (0.49)^a^	1.84 (0.72)	<0.001
Modified total CSVD score, mean (SD)	1.85 (0.7)^a^	1.75 (0.91)^a^	3.3 (1.12)	<0.001

PD, Parkinson’s disease; SD, standard deviation; UPDRS III, Part III of Unified Parkinson’s Disease Rating Scale; WMH, white matter hyperintensity; LI, lacunar infarction; CMB, cerebral microhemorrhage; PV, periventricular; BG-EPVS, basal ganglia-enlarged perivascular space; CSVD, cerebral small vessel disease. “^a^” means that compared with PIGD, *p* < 0.05.

### Univariate regression analysis for screening PIGD risk factors

The results of the univariate logistic regression analysis are presented in Table 2. According to the results, age (OR = 1.07, 95% CI = 1.021–1.122, *p* = 0.005), disease duration (OR = 1.336, 95%CI = 1.076–1.660, *p* = 0.009), UPDRS III (OR = 3.093, 95%CI = 2.144–4.462, *p* < 0.001), the presence of LI (OR = 3.064, 95% CI = 1.593–5.895, *p* < 0.001), total CSVD burden score (OR = 3.289, 95% CI = 1.904–5.680, *p* < 0.001), and the modified total CSVD burden score (OR = 4.930,95% CI = 3.032–8.018, *p* < 0.001) were potential risk factors for the PIGD subtype, while DM might be a protective factor (OR = 0.302, 95% CI = 0.104–0.875, *p* = 0.027) for PIGD.

**Table 2:** Univariate regression analysis for identifying the risk factors of PIGD.

Characteristics	OR (95% CI)	*p*
Demographics		
Male	0.605 (0.322,1.140)	0.128
Age	1.07 (1.021,1.122)	0.005
Education	0.911 (0.803,1.033)	0.146
Medical history		
Hypertension	0.639 (0.304,1.346)	0.239
Diabetes mellitus	0.302 (0.104,0.875)	0.027
Dyslipidemia	0.786 (0.333,1.854)	0.582
Smoking	0.896 (0.413,1.946)	0.782
Drinking	0.434 (0.167,1.131)	0.088
Clinical characteristics of PD		
Disease duration	1.336 (1.076,1.660)	0.009
UPDRS III	3.093 (2.144,4.462)a	<0.001
Imaging characteristics, median (IQR)		
Deep-WMH Fazekas score	1.224 (0.924,1.674)	0.150
Presence of LI	3.064 (1.593,5.895)	<0.001
Presence CMBs	1.615 (0.855,3.050)	0.140
PV-WMH Fazekas score,	1.152 (0.850,1.563)	0.362
BG-EPVS	1.005 (0.770,1.311)	0.971
Total CSVD burden score	3.289 (1.904,5.680)	<0.001
Modified total CSVD burden score	4.930 (3.032,8.018)	<0.001

PD, Parkinson’s disease; SD, standard deviation; UPDRS III, Part III of Unified Parkinson’s Disease Rating Scale; WMH, white matter hyperintensity; LI, lacunar infarction; CMB, cerebral microhemorrhage; PV, periventricular; BG-EPVS, basal ganglia-enlarged perivascular space; CSVD, cerebral small vessel disease.

### Multivariate regression analysis of the correlation between the severity of CSVD and PIGD

In the multivariate regression analysis adjusted for confounding factors such as gender, age, PD disease duration, alcohol consumption, diabetes, LI, CMBs, BG-EPVS and WMH (Model 2), the results showed that PIGD was independently associated with the total CSVD burden score (aOR = 7.16, 95% CI = 1.638–30.823, *p* = 0.009) and the modified total CSVD burden score (aOR = 6.034, 95% CI = 3.059–11.903, *p* < 0.001) (Table 3).

**Table 3:** Multivariate regression analyses for the severity of CSVD with PIGD.

Models	Total CSVD burden score		Modified total CSVD burden score	
	OR (95% CI)	*p*	OR (95% CI)	*p*
Unadjusted	3.289 (1.904,5.680)	<0.001	4.930 (3.032,8.018)	<0.001
Age and gender adjusted	7.061 (3.386,14.727)	<0.001	5.221 (3.104,8.782)	<0.001
Multivariable adjusted^*^	7.16 (1.638,30.823)	0.009	6.034 (3.059,11.903)	<0.001

CSVD, cerebral small vessel disease; OR, odds ratio; CI, confidence interval.

* adjusted for age, gender, diabetes mellitus, drinking, deep-WMH Fazekas score, presence of LI, presence CMBs, PV-WMH Fazekas score, and BG-EPVS.

To further validate the robustness of our multivariate logistic regression models, we assessed their goodness of fit and discriminatory power. The Hosmer-Lemeshow test indicated good calibration for both models: the age and gender adjusted model (total CSVD burden score: *χ*^2^ = 7.710, *p* = 0.462; modified total CSVD burden score: *χ*^2^ = 14.742, *p* = 0.064) and the multivariable adjusted model (total CSVD burden score: *χ*^2^ = 10.204, *p* = 0.251; modified total CSVD burden score: *χ*^2^ = 13.986, *p* = 0.082), with all *p*-values exceeding 0.05, suggesting no significant discrepancy between predicted and observed outcomes (Supplementary Table 6). Discriminatory ability, measured by the area under the receiver operating characteristic curve (AUC), was good for both models: 0.803 (95% CI: 0.737–0.870) and 0.880 (95% CI: 0.826–0.935) for the age and gender adjusted model and 0.865 (95% CI: 0.810–0.919) and 0.933 (95% CI: 0.896–0.971) for the multivariable adjusted model (Supplementary Table 7).

## 4 Discussion

This cross-sectional study provides robust evidence linking CSVD to the PIGD subtype in PD. Our key findings demonstrated that: This study shows that the incidence of PIGD is 49.07%, which is consistent with previous research reports (Chen et al., 2023; Luca and Rundek, 2015). The total CSVD burden score and the adjusted CSVD burden score of the PIGD subgroup patients were higher than those of the non-PIGD group patients. Even after adjusting for multiple confounding factors such as age, gender, and disease duration, the CSVD burden score and the adjusted CSVD burden score remained independent risk factors for PIGD. For every 1-point increase in the total CSVD burden score, the risk of PIGD increases to 7.16. And for every 1-point increase in the modified total CSVD burden score, the risk of PIGD increases to 6.034 times.

The onset of PD is intertwined with multiple brain circuitries, including those within the cortex, subcortex, and the limbic system (Jacob et al., 2023; Rosano et al., 2020). CSVD is characterized by WMH, LI, EPVS, CMBs, and brain atrophy as observed on magnetic resonance imaging (Pantoni, 2010). It may also contribute to neurological and psychiatric conditions, such as gait and cognitive dysfunctions (Chen et al., 2023). The total CSVD burden score provides an overall impression of cerebral small vessel pathologies, offering a more precise assessment of global cerebral small vessel damage. A growing body of research demonstrates a relationship between PD and CSVD. It appears that CSVD might be a risk factor for PD, while PD might exacerbate CSVD, and the two conditions might share some similar pathogenic mechanisms (Morley and Duda, 2012; Chen et al., 2021). Our findings align with previous research suggesting a vascular contribution to PD motor heterogeneity (Chen et al., 2023). The strong association between WMH and PIGD corroborates prior reports linking periventricular and frontal white matter damage to gait impairment in PD (Oveisgharan et al., 2021; Paolini Paoletti et al., 2021). The independent role of lacunes further supports involvement of strategic subcortical structures (e.g., basal ganglia, thalamus) critical for motor integration (Chen et al., 2021; Zhang et al., 2019). Notably, our use of a validated composite CSVD burden score extends beyond prior studies focusing on single markers, providing a more holistic assessment of vascular injury. The magnitude of association highlights CSVD as a major non-dopaminergic contributor to PIGD pathogenesis, potentially explaining the limited response of axial symptoms to levodopa.

The potential mechanism underlying the correlation between CSVD and PIGD remains unclear. However, we hypothesize that the following mechanisms may play a role in this process. Firstly, the integration of CSVD and PIGD may intensify the damage to the neural networks related to posture and gait control. WMH disrupts long-range frontal-striatal and frontal-pontine-cerebellar connections, impairing executive control of gait and balance (Morley and Duda, 2012; Wan H., et al., 2023; Wan S., et al., 2023). Lacunes in the thalamus or basal ganglia region may damage the basal ganglia-thalamus-cortex loop, exacerbating stiffness symptoms and postural gait disorders (Righart et al., 2013; Wardlaw et al., 2019). Secondly, the brain hypoperfusion induced by CSVD may accelerate the neurodegeneration in already vulnerable areas affected by PD (such as the substantia nigra, pedunculopontine nucleus, etc.), thereby exacerbating the symptoms of PD (Paolini Paoletti et al., 2021; Rosano et al., 2020). This synergy may manifest clinically as the PIGD phenotype, characterized by early falls, freezing, and poor response to dopaminergic therapy. Thirdly, CSVD can trigger chronic inflammatory responses, such as systemic inflammation caused by C-reactive protein and interleukin-6, and vascular inflammation induced by homocysteine, thereby damaging homeostasis and affecting dopaminergic neurons. This may, to some extent, trigger the occurrence of PIGD (Sanchez et al., 2025; Ma et al., 2023; Del Cuore et al., 2022; Wan H., et al., 2023; Wan S., et al., 2023).

Our study has provided epidemiological evidence for the correlation between the severity of CSVD and the occurrence of PIGD, emphasizing the importance of early screening and intervention for CSVD in patients with PD. Our study adds to the existing literature in several innovative ways. First, unlike previous studies that often focused on individual CSVD markers, we employed two validated composite scores to quantify the global CSVD burden, providing a more comprehensive assessment of vascular injury. Second, we specifically targeted the PIGD subtype, a clinically distinct and prognostically unfavorable phenotype, thereby addressing a gap in the literature regarding the vascular contributions to specific PD motor subtypes. Third, our findings strongly suggest that CSVD burden could serve as a valuable imaging biomarker to identify PD patients at the highest risk for developing debilitating axial motor impairments, which has significant implications for clinical prognostication and potential early intervention.

This study has several limitations that should be acknowledged. Firstly, the relatively small sample size from a single center limits the generalizability of the results. Multi-center large-sample studies are necessary to verify the findings. Secondly, the cross-sectional design cannot clarify the causal relationship between CSVD and PIGD. A longitudinal cohort study is needed to further verify the causal relationship between the two and the predictive effect of CSVD on PIGD. Thirdly, some confounding factors, such as detailed drug use status, were not included in the analysis, which may cause bias.

## 5 Conclusion

In conclusion, this study demonstrates that cerebral small vessel disease burden is an independent and robust correlate of the PIGD subtype in Parkinson’s disease, with severe white matter hyperintensities and lacunes being key contributors. The significant incremental predictive value of CSVD burden over clinical factors underscores its potential as a biomarker for identifying PD patients at the highest risk for debilitating gait and balance impairment. These findings highlight the convergence of vascular and neurodegenerative pathologies in shaping PD phenotypic heterogeneity. Clinically, our results advocate for the integration of vascular risk factor screening and brain MRI-based CSVD assessment into the management of PD patients, particularly those with early postural instability. Targeting vascular health may represent a viable strategy to modify the course of the PIGD subtype, offering hope for improving functional outcomes in this vulnerable population.

## Data Availability

The raw data supporting the conclusions of this article will be made available by the authors, without undue reservation.
