# Supplementary material for: Correlation between cerebral small vessel disease and postural instability/gait difficulty subtype in Parkinson’s disease patients
Source: Front Aging Neurosci. 2025 Dec 2;17:1686214. doi: 10.3389/fnagi.2025.1686214 (PMC12706657; doi:10.3389/fnagi.2025.1686214)
Supplement: Supplementary file 1 [file Table_1.docx]

Supplementary Material

# Supplementary Tables

**Supplementary** **Table 1.** Definition of cerebral small vessel disease (CSVD) subtypes

| CSVD subtypes | Definitions |
| --- | --- |
| White matter hyperintensities (WMH) | Evaluated based on T2 and Flair sequences by the Fazekas rating scale |
| Cerebral microbleeds (CMBs) | Defined as round or ovoid hypointense lesions measuring 2–10 mm in diameter on susceptibility-weighted imaging (SWI) |
| Lacunae | Characterized as small, fluid-filled cavities located in the cortex, displaying signal intensities similar to cerebrospinal fluid (CSF) on T1, T2, or FLAIR weighted images, with diameters ranging from 3 to 20 mm. |
| Enlarged perivascular spaces (EPVS) | Described as linear, round, or oval structures less than 3 mm in diameter, exhibiting CSF-like signal intensity. |

**Supplementary Table 2.** Fazekas scale scores

| Score | Periventricular White Matter Signal | Deep White Matter Signal |
| --- | --- | --- |
| 0 | No white matter lesions | No white matter lesions |
| 1 | Cap or pencil-thin subcortical lesions | Dot-like lesions |
| 2 | Smooth halo-like white matter lesions | Confluent lesions |
| 3 | Irregular periventricular high signal extending to deep white matter | Extensive confluent white matter lesions |

**Supplementary** **Table 3.** Basal ganglia and centrum semiovale perivascular spaces (PVS) Scores

| Grade | Basal Ganglia | Centrum Semiovale |
| --- | --- | --- |
| Grade 1 | <5 | Total white matter PVS <10 |
| Grade 2 | 5-10 | Total white matter PVS >10, but at the maximum level PVS <10 |
| Grade 3 | >10 but still countable | At the maximum level, PVS between 10-20 |
| Grade 4 | PVS difficult to count, basal ganglia area shows sieve-like changes | At the maximum level, PVS >20 |

Notes: The basal ganglia region was defined as the anatomical area between the superior border of the caudate nucleus and the inferior border of the anterior perforated substance on magnetic resonance imaging (MRI) sequences. It was further classified into the upper two-thirds and lower one-third based on their distinct vascular supply (using the third ventricle as a reference point; the lower one-third was identified below the third ventricle) for individual distribution counts. The highest score obtained represented the cumulative score for the entire basal ganglia region. The centrum semiovale region encompassed the white matter located between 5mm below the cortical surface and the level of the lateral ventricles on MRI sequences.

**Supplementary Table 4.** The scoring criteria for evaluating the total cerebral small vessel disease (CSVD) burden score (range 0-4)

| Domain | Criteria | Score |
| --- | --- | --- |
| LI | Presence of at least one cerebral lacunae infarct. | 1 |
| WMH | Periventricular irregular lesions extending into the deep white matter (Fazekas score 3), or early confluent deep white matter lesions (Fazekas score 2 or 3). | 1 |
| EPVS | Greater than 10 enlarged perivascular spaces (EPVS) at the basal ganglia level (Grades 2-4). | 1 |
| CMBs | Presence of at least one cerebral microbleed lesion. | 1 |

Abbreviations: LI: lacunae infarct; WMH:white matter hyperintensities; EPVS: enlarged perivascular spaces; CMB: cerebral microbleed.

**Supplementary** **Table 5.** The scoring criteria for evaluating the modified total cerebral small vessel disease (CSVD) burden score (range 0-6)

| Domain | Criteria | Score |
| --- | --- | --- |
| LI | Presence of at least one cerebral lacunae infarct. | 1 |
| WMH | Total score of Fazekas in the periventricular and deep regions of the brain of 3-4 | 1 |
| EPVS | Greater than 20 enlarged perivascular spaces (EPVS) at the basal ganglia level. | 1 |
| CMBs | Presence of at least 1-4 cerebral microbleed lesion. | 1 |
| CMBs | Presence of ≥5 cerebral microbleed lesion. | 2 |
| WMH | Total score of Fazekas in the periventricular and deep regions of the brain of 3-4 | 2 |

Abbreviations: LI: lacunae infarct; WMH:white matter hyperintensities; EPVS: enlarged perivascular spaces; CMB: cerebral microbleed.

**Supplementary** **Table 6.** The Hosmer-Lemeshow test results for model consistency

| Models | Total CSVD burden score | | Modified total CSVD burden score | |
| --- | --- | --- | --- | --- |
|  | χ² | *P* | χ² | *P* |
| Age and gender adjusted | 7.710 | 0.462 | 14.742 | 0.064 |
| Multivariable adjusted**^＊^** | 10.204 | 0.251 | 13.986 | 0.082 |

**Abbreviations:** CSVD, cerebral small vessel disease;

**Notes: ^＊^：**adjusted for age, gender, diabetes mellitus, drinking, deep-WMH Fazekas score, presence of LI, Presence CMBs, PV-WMH Fazekas score and BG-EPVS.

**Supplementary** **Table 7.** The area under the receiver operating characteristic curve (AUC) result for discriminatory ability

| Models | Total CSVD burden score | | | Modified total CSVD burden score | |
| --- | --- | --- | --- | --- | --- |
|  | | AUC | 95% CI | AUC | 95% CI |
| Age and gender adjusted | | 0.803 | 0.737-0.870 | 0.880 | 0.826-0.935 |
| Multivariable adjusted**^＊^** | | 0.865 | 0.810-0.919 | 0.933 | 0.896-0.971 |

**Abbreviations:** AUC, the area under the receiver operating characteristic curve; CSVD, cerebral small vessel disease; OR: odds ratio; CI: confidence interval;

**Notes: ^＊^：**adjusted for age, gender, diabetes mellitus, drinking, deep-WMH Fazekas score, presence of LI, Presence CMBs, PV-WMH Fazekas score and BG-EPVS.
